# Ethnicity Influences Risk of Inflammatory Bowel Disease (IBD)-Associated Colon Cancer: A Cross-sectional Analysis of Dysplasia Prevalence and Risk Factors in Hispanics and Non-Hispanic Whites With IBD

**DOI:** 10.1093/crocol/otab016

**Published:** 2021-05-04

**Authors:** Oriana M Damas, Gabriella Raffa, Derek Estes, Grechen Mills, David Kerman, Ana Palacio, Seth J Schwartz, Amar R Deshpande, Maria T Abreu

**Affiliations:** 1 Division of Gastroenterology, Department of Medicine, University of Miami Miller School of Medicine, Miami, Florida, USA; 2 Internal Medicine, Jackson Memorial Hospital/University of Miami Miller School of Medicine, Miami, Florida, USA; 3 Department of Public Health Sciences, University of Miami Miller School of Medicine, Miami, Florida, USA

**Keywords:** colon dysplasia, colon cancer, minorities, Hispanics, inflammatory bowel disease

## Abstract

**Background:**

Inflammatory bowel disease (IBD) is an emerging disease in Hispanics. In this study, we examine the prevalence of IBD-related colon dysplasia (IBD-dys) in Hispanics versus non-Hispanic whites (NHWs) and compare differences in established clinical and environmental risk factors.

**Methods:**

We performed a cross-sectional analysis on adult Hispanics and NHWs with IBD who met criteria for colorectal cancer surveillance and were followed at our center between 2008 and 2018. Clinical variables and IBD phenotype were recorded. Lifestyle IBD-dys risk factors were examined, including smoking and lack of physical activity. Using multivariable regression, we compared the prevalence of IBD-dys in Hispanics versus NHW, using relevant covariates. Receiver operating characteristic and area under the curve were performed to find the best fitting model.

**Results:**

A total of 445 IBD patients were included (148 Hispanics and 297 NHWs). IBD phenotype was similar between groups, except that Hispanics had shorter disease duration, a lower frequency of Crohn’s disease-related complications, and lower reported use of steroids. Frequency of surveillance colonoscopies was similar between Hispanics and NHW. There were no differences in median body mass index between Hispanics and NHW [26.5 (IQR 6.0) vs 25.0 (IQR 6.0), *P* = 0.40]. Hispanics were less likely than NHW to consume alcohol but smoking history was similar between groups. Three out of 148 Hispanic patients had IBD-dys (2.02%) compared to 29 out of 297 NHWs (9.76%). Adjusting for disease duration, primary sclerosing cholangitis, family history of colon cancer, and smoking, Hispanics had a lower prevalence of IBD-dys compared to NHW [OR_adjusted_ = 0.207 (95% CI 0.046–0.938), *P* = 0.008].

**Conclusions:**

Hispanics with IBD undergoing surveillance had a lower prevalence of IBD-dys than their NHW counterparts, despite similar risk factors. Future studies should examine dietary and microbial factors that may explain differences in risk.

## Lay Summary

We compared the prevalence of inflammatory bowel disease-related precancerous colon lesions between Hispanics and non-Hispanic whites (NHWs). We find that precancerous lesions occur more frequently among NHW despite accounting for established risk factors of colon cancer including disease duration and smoking history.

## BACKGROUND

Colorectal cancer (CRC) is the second leading cause of cancer in US Hispanics.^1^ Longstanding colitis from inflammatory bowel disease (IBD) is a known risk factor for CRC. Given that US Hispanics have only recently begun to be diagnosed with IBD, Hispanics with IBD may be especially at risk.^2^ Additionally, prior literature indicates that once CRC occurs, Hispanics with CRC have poorer outcomes than non-Hispanic white (NHW).^1^ It is therefore important to determine whether there is an increased risk of colon lesions (dysplasia) and CRC among Hispanics who develop IBD in order to develop surveillance strategies to minimize healthcare disparities.

The incidence of IBD-associated colon cancer (IBD-CRC) ranges from 1% to 10% in primarily European populations, and more recent studies indicate that IBD-CRC may be decreasing in ulcerative colitis (UC).^3^ Advancements in therapeutic strategies with more aggressive control of inflammation as well as improved endoscopic technology may explain a decreased CRC observed in European populations.^3,4^ However, we have yet to understand what the IBD-CRC risk is across nonwhite populations, and new data suggest that although CRC may be decreasing, the mortality of IBD-CRC is higher than in sporadic CRC.^3^ These patterns suggest that we still have more to learn about which populations are at risk for IBD-CRC and what underlying factors may be responsible for poorer outcomes.^3^ Known risk factors for development of IBD-CRC include presence of primary sclerosing cholangitis (PSC), extensive colon involvement, smoking history, poorly controlled inflammation, and longer duration of disease.^4^ Thus far, phenotypic studies examining IBD in Hispanics suggest that, although IBD is a new disease in this population, Hispanics share a similar prevalence of several risk factors including similar frequencies of pancolitis and PSC but may have a milder disease course.^5^ Therefore, it becomes of pressing need to study disease-related complications including IBD-CRC in nonwhite populations such as Hispanics.

Compared to other minorities in the United States, Hispanics are at increased risk for development of sporadic adenomas and for late-stage presentation of CRC.^6–9^ Interestingly, despite socioeconomic disadvantages and a higher reported rate of precancerous lesions, the incidence of sporadic, non-IBD-CRC is lower in Hispanics than in NHWs.^10^ This may not be the same for all Hispanics, however. A National Cancer Institute Surveillance Program in the United States performed between 1992 and 2013 indicated that Caribbean and South American Hispanics have a higher incidence of CRC compared to Central Americans.^11^ Differences by country of origin may be a result of varying nutrition patterns, access to medical care, and underlying genetic risk.^6–9,12–16^ Our study cohort captures the higher risk Hispanic populations but also evaluates the cohort of Hispanics with lower CRC risk as the South Florida Hispanic population is comprised of patients from the Caribbean, Central America, and South America.

Our primary objective was to conduct a cross-sectional analysis of the prevalence of IBD dysplasia in Hispanics compared to NHW in a cohort meeting criteria for surveillance. Secondary objectives were to compare demographic, clinical, and environmental risk factors for IBD-related colon dysplasia (IBD-dys) between Hispanics and NHW. The present study is important because it is the first to investigate the extent to which ethnicity may independently influence risk of IBD-dys and whether future prediction models should also factor this into account.

## METHODS

### Clinic Cohort

We conducted a cross-sectional analysis of patients attending a tertiary referral IBD center or the adjacent academically affiliated safety net GI clinic in Miami between January 2008 and December 2018. Colonoscopy biopsies from both institutions are sent to the same GI trained academic pathologists. All the patients in clinic are asked to be part of an IBD registry database and research studies. After consent is obtained, we capture demographic and clinical data from patients attending the clinic.

The diagnosis of IBD was confirmed by the attending physician at the time of clinic. IBD was confirmed using standard methods, including a combination of clinical, imaging, endoscopic, histologic, and surgical findings. Inclusion criteria for this study were: adult patients with confirmed IBD, with a self-identified ethnicity of either Hispanic or NHW, and who were followed in 1 of the 2 GI clinics. To minimize heterogeneity, only NHW patients who self-identified as white were included (for instance, all patients who identified as non-Hispanic black were excluded). We only included patients who met criteria for surveillance: patients with Crohn disease (CD) or indeterminate colitis who had ≥30% of the colon involved and ≥8 years of disease; UC patients with at least left-sided colitis (E2 or E3 by Montreal classification) and ≥8 years of disease; patients with any extent of colonic disease and duration of disease who had a diagnosis of PSC. We queried our IBD database all patients who met criteria for surveillance and ascertained cases of colon dysplasia or CRC recorded at the time of the initial clinic visit or on follow-up visits. We confirmed the diagnosis of colon dysplasia by review of the electronic medical record and review of the colonoscopy and histology reports. All records were reviewed independently by authors G.R., D.E., and O.D.

### Clinical Classifications

We recorded detailed clinical information pertaining to each patient’s IBD diagnosis and presence of risk factors contributing to development of colon dysplasia. Age at diagnosis, disease duration, IBD type (CD, UC, or indeterminate colitis), and presence of PSC were recorded. IBD phenotype and location of disease were recorded using the Montreal classification. We assessed for UC disease severity using the Truelove–Witts severity index. Family history of colon cancer was recorded.

### Lifestyle Risk Factors

We compared well-established lifestyle risk factors for CRC between Hispanics and NHW. Questions asked were part of the IBD questionnaire that patients completed on their first clinic visit; these include validated PhenX toolkit questions and questions obtained from the GEM study querying body mass index (BMI), smoking history, use of nonsteroidal anti-inflammatory drugs (NSAIDs) (aspirin and others), use of oral contraceptives, alcohol use, and physical activity (see full questionnaire, Supplementary materials).^17,18^ Physical activity was queried using a validated PhenX toolkit question that defines vigorous, moderate, and seldom/sedentary activity and then asks about typical physical weekly activity. Patients were also asked about their perceived physical activity relative to people of their own age.

### Colonoscopy Surveillance and Dysplasia Definitions

We recorded the date of the first colonoscopy showing colon dysplasia and, if available, the preceding colonoscopy date (when no dysplasia was found). Colonoscopy dates and intervals in between colonoscopies were recorded from the electronic medical record, including for procedures performed at our center and elsewhere. We classified individuals as having an “appropriate surveillance colonoscopy” if they met criteria for IBD-associated colon cancer surveillance (which included all patients in this cohort) and had received a colonoscopy within at least 3 years of the prior colonoscopy.

We recorded use of chromoendoscopy, number of biopsies, use of high-definition white light when available. Mayo score for UC and SES scores for CD were also recorded in those with available scoring. Given variability in the number of biopsies obtained for surveillance and in the use of chromoendoscopy for colonoscopies performed at our center versus at other GI practices, we used interval of colonoscopy as a surrogate for appropriate screening. We determined no more than 3 years in between colonoscopies was appropriate, based on available recommendations and prior studies.^13,19–22^ For patients with PSC, we considered an annual colonoscopy an appropriate interval.

In all patients reporting a history of IBD-dys or CRC, prior endoscopy and histology reports were reviewed; all patients with reported dysplasia had prior records available. We excluded any patient with inconclusive findings (ie, indefinite dysplasia or atypia). We defined colon dysplasia as IBD-dys if the endoscopic and histologic findings of the area within that same colon segment had evidence of prior or present inflammation. By corollary, we identified colon dysplasia as “sporadic” if there was no endoscopic or histologic evidence of prior or present inflammation in that segment of colon. All endoscopy and histology reports were reviewed independently by authors G.R., D.E., and O.D., and disagreement was adjudicated by repeat evaluation of those patients’ records to determine whether inflammation was ever present in the relevant colon segment (possibly missed on initial evaluation due to the possibility of complete mucosal healing). In 1 patient, the determination of sporadic versus IBD-related dysplasia was still unclear, so adjudication was done after conferring with the treating physician. All diagnoses of dysplasia were reviewed by 2 expert GI pathologists at our institution (a total of 31 cases) with the exception of 1 patient. Biopsies read included those with colonoscopies performed at other GI centers that were then sent to be read at our institution for confirmation of dysplasia. Only 1 patient did not have biopsies read by a GI pathologist (to our knowledge) but he was kept in the analysis because had a history of colon cancer and we felt that a histologic finding of colon adenocarcinoma has less interobserver variability and this finding was also confirmed again by surgical specimen.

### Main Outcomes

Our main outcome was prevalence of IBD-dys and IBD-CRC in Hispanics compared to NHW. Secondary outcomes were comparisons of known clinical and environmental risk factors for IBD-dys and appropriate surveillance intervals between Hispanics and NHW with IBD.

### Statistical Analysis

Demographics, IBD phenotype, and environmental exposures were compared between Hispanics and NHW using chi-square and analysis of variance. For non-normal distributions (e.g duration of disease), we performed a Mann–Whitney to test for significant group differences. We compared prevalence of IBD-dys between Hispanics and NHW using chi-square analyses. We then performed multivariable binary logistic regression (MVR) to compare the prevalence of IBD-dys across groups, taking into account known risk factors for dysplasia and variables that reached significance in univariate analysis. We evaluated significance of the covariates in the model and of each independent variable as an interaction with ethnicity. Independent variables were centered prior to computing interaction terms. We then used a multistage decision process for retaining and dropping covariates, using stepwise regression modeling and incorporating variables with *P* values ≤0.10. Goodness-of-fit testing for MVR models was performed using receiver operating characteristic (ROC) curves and estimates of the area under the curve (AUC). Differences in surveillance colonoscopies were also examined between Hispanics and NHW using Student *t* tests. Statistical analyses were performed using SAS Studio.

### Study Oversight

The present study was approved by the University of Miami Miller School of Medicine Institutional Review Board.

## RESULTS

### Demographic Characteristics in Hispanics and Non-Hispanics

**Figure 1.**
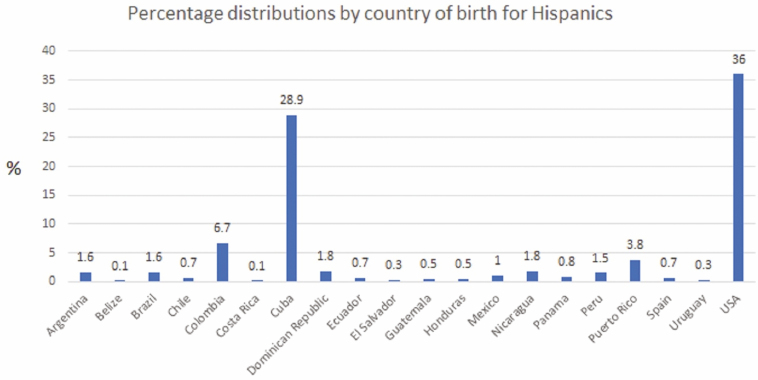
Country of birth of Hispanics in our cohort.

A total of 2781 IBD patients were queried from the IBD database, of whom 445 met criteria for surveillance: 148 Hispanics and 297 NHWs. Our population of Hispanics comprised primarily Cuban-born participants (28.9%), then US-born participants of Cuban-born parents (36%) followed by Colombian-born (6.7%) and Puerto Rican-born (3.8%) participants. The rest of the Hispanics came from 16 other countries (Fig. 1). Most NHWs were born in the United States (78.2%), followed by Canada (1.5%). Other countries each represented 0.1%–0.5% of the NHW cohort and were primarily European. Duration living in the United States was similar between Hispanic and NHW immigrants [*M* 26.31 (SD 16.2) vs *M* 27.3 (SD 16.1), *P* = 0.74, respectively]. Hispanics were less likely than NHW to have a college education (49% vs 62.5%, respectively) [χ ^2^ (4, N = 445) = 15.48, *P* = 0.004].

Hispanics had an older age at IBD diagnosis [median 29.52 (IQR 22.5) vs 23.56 years (IQR 17.9)] (Mann–Whitney *U* = 24,743, *P* = 0.02) and a shorter duration of disease [median 14.0 (IQR 14.0) vs 16.0 (IQR 14.0) years] (Mann–Whitney *U* = 18,956.0, *P* = 0.023) compared to NHW. Hispanics also had a lower prevalence of other autoimmune diseases compared to NHW (6.8% vs 14.9%, respectively) [χ ^2^ (2, N = 445) = 6.19, *P* = 0.045]. Age at time of initial or last clinic visit did not differ between Hispanics and NHW (Table 1). Family history of colon cancer, number of comorbidities, and diagnoses of other cancers were similar between groups (Table 1). Hispanics and NHW were followed in clinic for a similar duration of time [mean 1.73 (SD 2.08) vs 1.39 years (SD 1.99), *t*(476) = 1.69, *P* = 0.104]. The majority of patients including Hispanics and NHW had health insurance: HMOs, group health plans, Medicare or Medicaid. However, a greater percentage of NHW (81.72%) reported having private insurance compared to Hispanics (62.76%) and there was a slightly higher percentage of Hispanics patients without insurance (6.9%) compared to NHW (3.10%), *x*^2^(4) = 22.3, *P* = 0.0002. The majority of patients (366 patients) attended the tertiary referral clinic and the remaining 79 patients attended the safety net community GI clinic.

**Table 1.** Demographics and Environmental Exposures in Hispanics Compared to NHWs

Variables	Hispanics (n = 147)	Non-Hispanic (n = 297)	*P*
Age of diagnosis (median, IQR)	29.52 (22.54)	23.56 (17.98)	**0.022**
Age at last visit (median, IQR)	48.00 (23.00)	46.00 (26.00)	0.470
Age of first clinic visit (median, IQR)	46.10 (22.32)	43.99 (24.51)	0.434
Duration of disease (median, IQR)	14.00 (14.00)	16.00 (14.00)	**0.023**
Smoking history			0.64
Ex-smoker	38 (25.9%)	64 (21.5%)	
Never smoked	102 (69.4%)	214 (72.1%)	
Current smoker	7 (4.8%)	18 (6.1%)	
Family history of IBD	29 (20.7%)	87 (31.4%)	**0.021**
Family history of colon cancer	0 (0%)	4 (1.3%)	0.307
Family history of non-GI cancers	9 (6.1%)	30 (10.4%)	0.193
BMI	26.50 (6.0)	25.00 (6.0)	0.40
Presence of comorbidities			0.083
1	0 (0%)	3 (2.0%)	
2	1 (0.3%)	0 (0%)	
3+	1 (0.3%)	2 (0.7%)	
Physical activity			0.58
Moderately active (at least 3 times per week)	58 (39.4%)	121 (40.6%)	
Seldom active, preferring sedentary activities	50 (34.0%)	91 (30.8%)	
Vigorously active for at least 30 min, 3 times per week	38 (26.1%)	84 (28.2%)	
Activity compared to others of same age			0.35
Much less active	10 (7.0%)	28 (9.6%)	
Less active	39 (26.5%)	70 (23.5%)	
About as active	51 (34.7%)	107 (35.9%)	
More active	30 (20.2%)	60 (20.3%)	
Much more active	17 (11.3%)	32 (10.7%)	

In bold are *p*-values for associations that reached statistical significance (*P* < .05).

### IBD Phenotypes and Frequency of Surveillance Colonoscopies Do Not Differ Between Hispanics and NHW

Most patients in our cohort who met criteria for surveillance had UC (Table 2). A similar percentage of Hispanics and NHW had pancolitis (either in UC or CD) [75.5% vs 75.6%, χ ^2^ (1, N = 445 = 0.98, *P* = 1.0)]. Hispanics had fewer surgeries for CD-related complications than NHW [2.0% vs 9.2%, χ ^2^ (2, N = 285 = 6.67, *P* = 0.036)] but that was not the case for UC. There were no other differences in phenotypic characteristics of IBD between Hispanics and NHW, including presence of PSC, location of colonic disease in UC or CD, or disease behavior for CD (inflammatory vs stricturing vs fistulizing) (Table 2). Hispanics and NHW had similar mild to moderate severity of UC (S0 and S1 disease by Truelove–Witts index) (*P* = 0.190) (Table 2). There was no difference in frequency of IBD-related hospitalizations between Hispanics and NHW, including hospitalizations for flares, obstructions, and surgery-related complications (data not shown).

**Table 2.** Clinical Characteristics and Disease Phenotype of Hispanics and Non-Hispanics With IBD

	Hispanics (n = 147)	Non-Hispanics (n = 297)	*P*
IBD type			
CD	46 (31.3%)	105 (35.4%)	0.456
UC	101 (68.7%)	192 (64.6%)	
Pancolitis (CD or UC)	111 (75.5%)	223 (75.6%)	1.00
PSC	19 (13.5%)	30 (10.3%)	0.335
Disease behavior by Montreal classification			
Location of disease of CD			
L1: ileal disease	2 (4.3%)	0 (0%)	0.085
L2: colonic	11 (23.9%)	31 (29.5%)	
L3: ileocolonic	33 (71.7%)	74 (70.5%)	
L4: upper GI tract	0 (0%)	0 (0%)	
Behavior of CD			
Inflammatory	23 (54.8%)	56 (55.4%)	0.891
Stricturing	13 (31%)	28 (27.7%)	
Penetrating/fistulizing	6 (14.3%)	17 (16.8%)	
Perianal disease	21 (51.2%)	35 (38.7%)	0.187
Location of disease UC			
Pancolitis	70 (69.3%)	122 (63.9%)	0.523
Left-sided disease	31 (30.7%)	68 (35.6%)	
Proctitis	0 (0%)	1 (0.5%)	
UC disease severity			
S0	3 (2.0%)	4 (1.3%)	0.175
S1	43 (29.3%)	58 (19.5%)	
S2	40 (27.2%)	98 (33.0%)	
S3	16 (10.9%)	30 (10.1%)	
Medications (ever use)			
5-Aminosalicylates	45 (30.6%)	97 (33.7%)	0.589
Immunomodulators	45 (30.6%)	118 (41.0%)	**0.022**
Cyclosporine	1 (0.7%)	15 (5.2%)	**0.011**
Steroids	105 (72.9%)	242 (85.2%)	**0.003**
Biologics (anti-TNFs, ustekinumab and vedolizumab)	50%	52.3%	0.682
Appropriate* surveillance colonoscopy	39 (95.1%)	96 (93.2%)	0.51

In bold are p-values for associations that reached statistical significance (*P* < .05).

*Based on available data in a total of 144 patients.

Hispanics and NHW had a similar frequency of biologic use (50% and 52.3%, respectively) [χ ^2^ (1, N = 423) = 0.206, *P* = 0.682]. Similarly, there was no difference between Hispanics and NHW in frequency of 5-aminosalicylate use. However, Hispanics had lower percentages of corticosteroid, immunomodulator, and cyclosporine use compared to NHW (Table 2). For example, 72.9% of Hispanics reported ever using steroids compared to 85.2% of NHW, χ ^2^ (1, N = 428) = 9.41, *P* = 0.003 (Table 2). Appropriate surveillance colonoscopy, defined as at least 1 every 3 years, was no different between Hispanics and NHW; a total of 95.1% of Hispanics and 93.2% of NHW had appropriate surveillance colonoscopy screening [χ ^2^ (1, N = 144) = 0.184, *P* = 0.51].

### Environmental Risk Factors for CRC Are Similar Between Hispanics and NHWs

We examined several environmental risk factors for CRC between Hispanics and NHW. We found that both Hispanics and NHW were overweight (defined as a BMI ≥25) and that there were no differences in BMI between Hispanics and NHW [median 26.5 (IQR 6.0) vs median 25.0 (IQR 6.0)], respectively, *U* = 20,390.50, *P* = 0.40. Hispanics and NHW also had a similar prevalence of current and prior smoking (Table 1). However, Hispanics were less likely to consume alcohol in the last year than NHW (17% vs 30.6%, respectively [χ ^2^ (3, N = 435) = 11.252, *P* = 0.01].

Hispanics reported a lower current use of NSAIDs than NHW (8.2% of Hispanics reported use compared to 9.7% of NHW [χ ^2^ (2, N = 435) = 20.80, *P* = 0.04]. There was no difference in use of oral contraceptives between Hispanic and NHW women (23.8% of Hispanic women reported prior use compared to 25.3% of NHW women, *P* = 0.40). There were no differences in frequency of physical activity between Hispanics and NHW [χ ^2^ (3, N = 445) = 1.950, *P* = 0.58]. A total of 39.4% of Hispanics and 40.6% of NHW, reported being moderately active at least 3 times per week, which was described as “activity where your heart beats faster than normal (i.e., fast walking, aerobics class, strength training, swimming gently).” In this same fashion, there were no differences between Hispanics and NHW in perceived physical activity compared to others their age. In both groups, a greater percentage reported to be “about as active” as people their own age (34.7% of Hispanics and 35.9% of NHW) [χ ^2^ (5, N = 435) = 5.61, *P* = 0.35; Table 1].

### The Prevalence of IBD-Associated Dysplasia (IBD-Dys) and IBD-Related Colon (IBD-CRC) Is Lower in Hispanics in Multivariable Analysis

**Figure 2.**
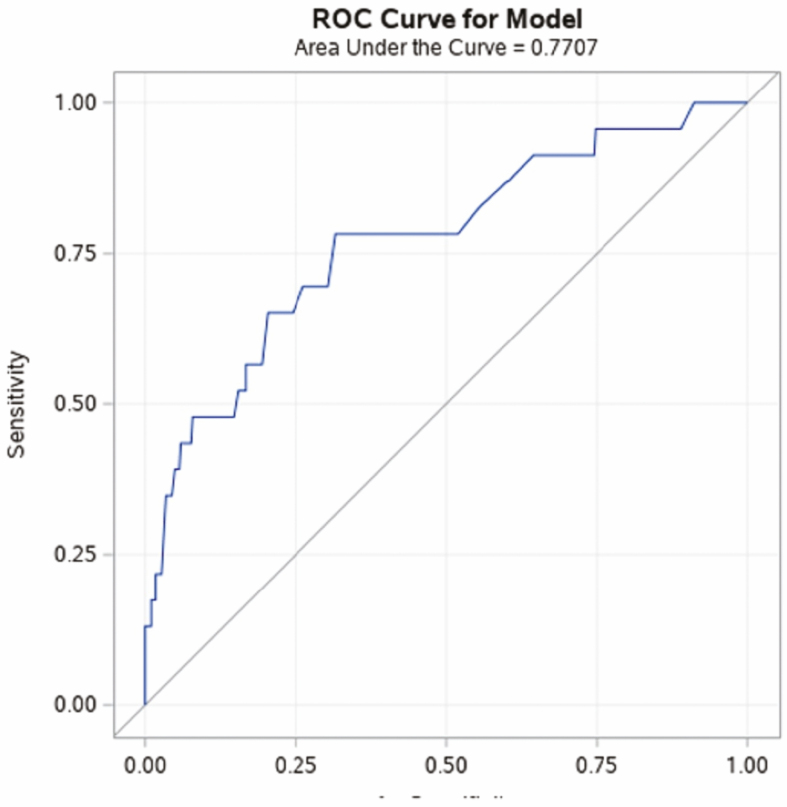
ROC and AUC for stepwise binary logistic regression model examining the influence of ethnicity, duration of disease, presence of PSC, and family history of colon cancer on IBD dysplasia.

Three Hispanic patients out of 148 had dysplasia (2.02%) (either IBD-dys or IBD-CRC). In contrast, 29 NHWs out of 297 had dysplasia (9.76%) (IBD-dys or IBD-CRC). Two (0.42%) Hispanics and 22 (7.4%) NHW had IBD-dys, χ ^2^ (1, N = 445) = 7.14, *P* = 0.008. A fewer percentage of Hispanics versus NHW had IBD-CRC, although this did not reach statistical significance [1 (0.7%) vs 7 (2.4%), χ ^2^ (1, N = 445) = 1.60, *P* = 0.28]. Sporadic adenomas were observed in 2 Hispanics and 8 NHWs (data not shown). We used multivariable logistic regression models to examine the influence of ethnicity on presence of any dysplasia (IBD-dys and IBD-CRC), taking into account interaction and confounder variables. We incorporated covariates in the stepwise regression model that had statistical significance of at least 0.1 in the univariate analyses. Independent variables that were significantly different between Hispanics and NHW on univariate analyses were duration of disease, alcohol use, and family history of IBD. Variables that independently influenced IBD-dys (family history of colon cancer and presence of PSC) were also included in the model. There was no interaction between independent variables and ethnicity. In stepwise regression, ethnicity, duration of disease, presence of PSC, and family history of colon cancer were retained in the model (Table 4). In the multivariable regression analysis, Hispanics had a lower likelihood of developing dysplasia (including precancerous and cancer lesions) than NHW [OR_adjusted_ 0.207 (95% CI 0.046–0.938]. A ROC curve for the model was created with an AUC of 0.77 (Fig. 2).

**Table 4.** Multivariable (Stepwise) Binary Logistic Regression Model Examining the Influence of Ethnicity on IBD-Associated Dysplasia, Accounting for Relevant Dysplasia Risk Factors

MVR Model, *P* 4.69 × E^−41^	*B*	Standard Error	DF	Significance	OR	95% CI
Ethnicity (ref = non-Hispanic)	−1.585	0.763	1	0.038	0.207	0.046–0.914
Duration of disease	0.0529	0.0161	1	0.015	1.054	1.022–1.088
Family history of colon cancer (ref = 0)	3.2522	1.346	1	0.025	25.85	1.847–361.761
Presence of PSC (ref = 0)	1.411	0.566	1	0.015	4.011	1.303–12.433

### Endoscopic and Histologic Characteristics of IBD-Associated Dysplasia and CRC in Hispanics and NHWs

Dysplasia occurred more frequently in UC than in CD in both Hispanics and NHW (Table 3). There was no difference in location of colon dysplasia between Hispanics and NHW (Table 3). When comparing endoscopic techniques including use of chromoendoscopy and high-definition white light, we found no differences in the use of these techniques between Hispanics and NHW. A total of 58.33% of NHW had colonoscopies done with chromoendoscopy compared to 41.67%, of Hispanics, *x*^2^(1) = 0.067, *P* = 0.79. All 400 patients with colonoscopies performed at our center were done with high-definition white light endoscopies. In these analyses, there was no difference in use of HD WL between Hispanics and non-Hispanics. We also recorded number of biopsies taken during colonoscopies done at our institution. In these, we find that on average, a greater number of biopsies (including random plus targeted biopsies) were done in colonoscopies done in NHW compared to those in Hispanics [avg. 21.05 (SD 9.39) and 17.78 (SD 9.38), *t*(219) = −2.42, *P* = 0.02]. On univariate analyses, we find that presence of UC and Mayo score also influenced number of biopsies taken (*P* < 0.0001 and *P* = 0.03). There was no significant association between duration of disease and number of biopsies (*P* = 0.07). After multivariable regression analysis incorporating presence of UC, Mayo score, and duration of disease, we find that there was no longer a relationship between number of biopsies taken and ethnicity (*F*(1) = 0.78, *P*_ethnicity_ = 0.38).

**Table 3.** Location of Dysplasia and Histology of Dysplasia in Hispanics and Non-Hispanics

	Hispanics (n = 3)	Non-Hispanics (n = 29)	*P*
Prevalence of dysplasia in UC	3 (100%)	26 (89.65%)	0.077
Prevalence of dysplasia in CD	0 (0%)	3 (10.3%)	
Location of colonic IBD dysplasia			
Cecum	0 (0%)	3 (10.3%)	1.00
Ascending	1 (33.3%)	13 (44.8%)	1.00
Transverse	0 (0%)	6 (20.7%)	1.00
Descending	1 (33.3%)	7 (24.1%)	1.00
Sigmoid	0	5 (17.23%)	1.00
Rectum	1 (33.3%)	0	1.00
IBD-associated severity of dysplasia			
Low-grade dysplasia	1 (33.33%)	17 (58.6%)	0.009
High-grade dysplasia	1 (33.33%)	5 (17.24%)	0.057
Adenocarcinoma	1 (33.33%)	7 (24.13%)	0.279
Endoscopic characteristics of IBD-associated dysplasia			
Visible dysplasia	2 (66.67%)	21 (72.41%)	0.019
Invisible dysplasia	1 (33.33%)	8 (27.59%)	
Polypoid	2 (66.67%)	21 (72.41%)	0.050
Non-polypoid/sessile	1 (33.33%)	8 (27.59%)	
Distinct borders	2 (66.67%)	13 (44.82%)	0.050
Endoscopically resectable	2 (66.67%)	17 (58.62%)	0.042
Surgery done for IBD dysplasia	1 (33.33%)	12 (41.38%)	0.021

Endoscopic disease severity graded by Mayo score in UC and simple endoscopic score for Crohn disease (SES-CD) score in CD was not different between Hispanics and NHW. We find that the mean SES-CD of colonoscopies in Hispanics was 5.39 compared to the SES-CD of colonoscopies performed in NHW 7.0, *t*(42) = −0.63, *P* = 0.53. Similarly, most Hispanics (58.33%) and NHW (60.27%) were in remission (Mayo 0) [*x*^2^(3) = 0.16, *P* = 0.98]. On histology, low-grade dysplasia was the most common histologic diagnosis among NHW. Hispanics had equal frequencies of low-grade dysplasia, high-grade dysplasia, and CRC (although sample sizes were small). Most dysplasia was visible endoscopically and polypoid appearing in both Hispanics and NHW (Table 3). We find that a greater percentage of NHW had surgical resection for dysplasia compared to Hispanics despite having lower grade dysplasia, although this is hard to interpret given the low frequency of dysplasia in Hispanics [12 (41.3% in NHW vs 1 (33.3%) in Hispanics, *P* = 0.021].

## DISCUSSION

The current study is the first to examine ethnicity as a potential risk factor for IBD-associated dysplasia. We find that the prevalence of IBD-associated dysplasia (including both precancerous and cancerous lesions) is lower in Hispanics (2.02%) compared to NHW (7.4%) [OR_adjusted_ 0.207 (95% CI = 0.046–0.938)]. The prevalence of CRC in our cross-sectional analysis was also lower in Hispanics (0.2%) than in NHW (2.4%). Recently reported prevalence data on IBD-related CRC in Europeans ranged from 1% to 10%, which falls in line with the CRC prevalence data from our NHW participants. We compared potential underlying causes for differences in prevalence observed by ethnicity and found that Hispanics and NHW had similar frequencies of several established clinical, and lifestyle risk factors for dysplasia as well as similar endoscopic techniques and frequency of surveillance colonoscopies, thereby suggesting that these risk factors alone do not explain the observed differences in IBD prevalence. Our findings highlight important gaps in our understanding of dysplasia risk in IBD and in ethnic differences in particular; this suggests a need to personalize surveillance strategies by individual risk including ethnicity.

In this cross-sectional analysis, we find that risk for CRC and precancerous lesions is low in Hispanics. Interestingly, this pattern is despite notable disadvantages for Hispanics such as lower education level, a reported lower use of NSAIDs, and mostly similar clinical and/or lifestyle risk factors, such as a high prevalence of obesity and similar frequencies of weekly physical activity compared to NHW. Additionally, family history of colon cancer, presence of PSC, presence of pancolitis, disease severity, and history of present or prior smoking are similar between Hispanics and NHW, indicating that these established CRC risk factors do not explain differences in prevalence of dysplasia observed. Unlike prior studies examining US Hispanics, we did not find evidence of differences in access to care between Hispanics and NHW, measured by presence of insurance, duration of follow-up, and frequency of appropriate surveillance colonoscopies.^9,23–25^ Although we think this may be a reflection of a predominantly insured cohort, this cohort works to our advantage because it allows us to categorize true prevalence not skewed by obvious healthcare disparities.

In our study, we found that Hispanics had a few protective factors for IBD-dys, including lower alcohol consumption.^26,27^ Another potential protective factor was a likely milder disease course. Hispanics had lower use of steroids and immunomodulators. Although Hispanics and NHW had a similar likelihood of being on biologics (including TNF inhibitors, ustekinumab, and vedolizumab), we found that NHWs were more likely to be on dual therapy with a biologic and an immunomodulator and were more likely to have ever been placed on steroids for IBD. We also found that NHW had more CD-related surgeries than Hispanics, all of which suggests a more complicated disease course in NHW. Although NHW had a longer disease duration with greater opportunity to develop these complications, our group has previously found in retrospective cohort studies that Hispanics, particularly foreign born, have a less severe disease phenotype of IBD including an older onset of disease, a lower prescription of biologics, and a lower hospitalization rate.^5^ We assessed UC disease severity using the Truelove and Witts disease severity index and found no differences in historical severity between Hispanics and NHW, but this measure has limitations for which a more accurate measurement of cumulative inflammatory burden needs to be developed.^28^ Our data therefore suggest that Hispanics may have fewer complications of IBD and possibly milder disease that could explain a lower frequency of IBD-dys, although longitudinal studies are needed to validate this finding with better measures of cumulative inflammatory burden. If the cumulative inflammatory burden is lower in Hispanics, then an exploration of nutritional versus genetic influencers is warranted.

The results of our study mirror CRC trends between Hispanics and NHW in the US population. Prior studies show that, although Hispanics may have a higher risk of sporadic tubular adenomas and greater health disparities in the United States, they have a lower incidence of CRC than NHW (29.8 per 100,000 vs 35.2 per 100,000).^29–31^ Our study therefore adds to the existing US literature indicating that Hispanics not only have a lower incidence of sporadic CRC but also of IBD-related CRC. These findings highlight the need to investigate what are the genetic and lifestyle underpinnings that explain ethnic differences in colon dysplasia prevalence. There are currently ~95 established genome-wide colon cancer genetic variants identified in European white populations but these variants have not been tested in Hispanic populations.^32^ Besides genetics, dietary and environmental behaviors protecting Hispanics from developing colon cancer may be at play.^33^ We know that the native “Hispanic” diet tends to be more “protective,” with higher fruits, vegetables, and lower red meat and this is the case in many Latin American countries.^34^ It is possible that this healthier diet may confer protection against CRC for a period of time even following immigration to the United States, although for how long remains elusive.

We also find in our study that once precancerous colonic lesions develop, there are overall similar endoscopic features including percentage of polypoid appearing dysplasia and endoscopic resectability. Due to low frequencies of CRC we are not able to interpret differences in stages of CRC diagnoses between Hispanics and NHW, but existing literature on sporadic CRC suggest that Hispanics have poorer outcomes once CRC develops.^1^ This may be due to poor access to care and differences in response to existing treatments available.^1^ We extrapolate from these data that, although Hispanics have lower frequencies of IBD-dys, once CRC develops Hispanics could still end up with worse outcomes than NHW. Larger studies are needed to confirm this hypothesis specifically in the IBD population.

Our study should be considered in light of several limitations. As a cross-sectional analysis, we were not able to assess for time to events, including time from disease onset to dysplasia. Due to limited records with exact dates, we decided to examine cross-sectional prevalence of dysplasia and existent risk factors. Additionally, our lifestyle questionnaire was based on self-report and was therefore subject to inherent biases including under or over reporting of behaviors (such as alcohol consumption and physical activity, respectively). We do, however, believe that our findings provide an important starting point for examining several risk exposures in the Hispanic IBD population. Further, Hispanics had a shorter duration of disease than NHW, which introduces lead-time bias. However, we took several steps to ensure that our findings could not be attributed to shorter disease duration. First, we examined only a cohort of patients who were considered “at risk” due to either presence of PSC or who had disease duration for at least 8 years; this minimized variability in duration of disease by ethnicity. Secondly, we performed a stepwise regression analysis taking into account duration of disease and we also examined separately duration of disease as an interaction variable with ethnicity. We found that although duration of disease contributed to the MVR, it did not attenuate the influence of ethnicity on dysplasia risk (Table 4).

Recent studies also call into question whether duration of disease is the most influential factor in determining dysplasia risk. There is growing evidence that cumulative inflammatory colonic burden, rather than disease duration, may best predict dysplasia risk.^35^ In our MVR model, using ROC/AUC goodness-of-fit measures, we found that the best predictors for colon dysplasia were ethnicity, disease duration, presence of PSC, and family history of colon cancer. However, this model still has a 23% chance to not predict dysplasia cases (AUC 77.1%, Fig. 2). Our current established risk factors for dysplasia, including duration of disease, remain imprecise as measures of dysplasia risk, so future studies should attempt to develop a cumulative inflammatory burden score and incorporation of “-omic” data to improve predictability of dysplasia.

Lastly, the Hispanic population is composed of individuals of diverse genetic and cultural backgrounds. As part of our research database, we ascertain country of birth of participants and their parents and grandparents, providing us with a detailed background on patient ancestry. Our population of Hispanics is mostly representative of a Cuban/Caribbean population, which limits some of the heterogeneity brought on by various countries of origin that could impact results (Fig. 1). Further, although a heterogenous group, Hispanic cultures still share similar cultural elements including dietary patterns as well as sociocultural aspects that could influence health risks and behaviors similarly.^36^

In conclusion, our study provides provocative data indicating that ethnicity may influence risk of IBD-dys. Hispanics develop less IBD-related dysplasia perhaps as a result of milder disease, although cumulative inflammatory colonic burden is not well characterized. Future studies should examine genetics and dietary habits as potential influences of a lower risk of IBD-dys among Hispanics. The present study serves to highlight ethnic disparities in prevalence of IBD-dys and highlights gaps in knowledge about influential predictors of IBD-related dysplasia.

## Supplementary Material

otab016_suppl_Supplementary_Materials

## Data Availability

Data are obtained from the IBD biorepository and REDCap database of the Crohn’s and Colitis Center. Data are available upon reasonable request.

## Notes

Funding: O.M. Damas: NIDH NIDDK 5K23DK117054-02, Global Studies in IBD and University of Miami Sylvester Cancer Center.

Conflict of Interest: O.M. Damas has a Pfizer research grant, prior speaking roles sponsored by PRIME Continuing Medical Education, participated in a Pfizer IBD Fellow Selections Committee. G. Raffa, D. Estes, A. Palacio, and Seth J. Schwartz: no conflict to declare. D. Kerman: consulting for Cleveland Clinic, teaching and lecturing for the Academy for Continued Healthcare Learning, teaching and lecturing for PRIME Continuing Medical Education, consulting for AbbVie. A.R. Deshpande has a Takeda research grant and is on the GI Health Foundation Advisory Committee, he also an ABIM committee member. M.T. Abreu reports grants and personal fees from Prometheus laboratories, Takeda Pharmaceuticals, Pfizer Inc., personal fees from Focus Medical Communications, Boehringer Ingelheim Pharmaceuticals, Gilead, Imedex, Cornerstones Health, Inc., Landos Biopharma, AbbVie Inc., Janssen Pharmaceuticals, Ferring Pharmaceuticals Inc., Allergan Inc., Amgen Inc., Celltrion Healthcare Co. Ltd, Millennium Pharmaceuticals, Inc., Theravance Biopharma Inc., Seres Therapeutics, Inc., Shire, UCB Biopharma SRL, outside the submitted work.
