# Supplementary material for: Ethnicity Influences Risk of Inflammatory Bowel Disease (IBD)-Associated Colon Cancer: A Cross-sectional Analysis of Dysplasia Prevalence and Risk Factors in Hispanics and Non-Hispanic Whites With IBD
Source: Crohns Colitis 360. 2021 May 4;3(2):otab016. doi: 10.1093/crocol/otab016 (PMC8924904; doi:10.1093/crocol/otab016)
Supplement: otab016_suppl_Supplementary_Materials [file otab016_suppl_supplementary_materials.docx]

*the following to be completed by patient. please ask your physician if you need assistance*

**Name:____________________________ First Language: ________________________**

**Gender** ○ Male ○ Female **Date of Birth:** ______________

*mm/ dd/ yyyy*

**City/Country of Birth:** _____________________________

**Date moved to the United States** (if not born in U.S.A)__________________________________________________________
 (if only remember the year please enter 01/01/YYYY)

**Ethnicity**: ○ Hispanic or Latino ○ Not Hispanic or Latino

**Race**: ○ Caucasian ○ Native American ○ Black/AfricanAmerican

○ Asian ○ native Hawaiian or Pacific Islander ○ More than one race, not defined ○ Unknown or I prefer not to answer

**Jewish:** ○ Yes ○ No ○ Unknown **If yes:**  ○Ashkenazi ○Sephardic ○ Other Jewish Ancestry

**Information about your parents:**

*Mother*: Where was she born (City/Country)?______________________________

**Ethnicity**: ○ Hispanic or Latino ○ Not Hispanic or Latino

**Race**: ○ Caucasian ○ Native American ○ Black/AfricanAmerican ○ Asian ○ native Hawaiian or Pacific Islander

○ More than one race, not defined ○ Unknown or I prefer not to answer

**Jewish:** ○ Yes ○ No ○ Unknown **If yes:**  ○Ashkenazi ○Sephardic ○ Other Jewish Ancestry

*Father:*  Where was he born (City/Country)?_______________________________

**Ethnicity**: ○ Hispanic or Latino ○ Not Hispanic or Latino

**Race**: ○ Caucasian ○ Native American ○ Black/AfricanAmerican ○ Asian ○ native Hawaiian or Pacific Islander

○ More than one race, not defined ○ Unknown or I prefer not to answer

**Jewish:** ○ Yes ○ No ○ Unknown **If yes:**  ○Ashkenazi ○Sephardic ○ Other Jewish Ancestry

**Information about your grandparents:**

*Maternal Grandmother*: Where was she born (City/Country)?_______________________

**Ethnicity**: ○ Hispanic or Latino ○ Not Hispanic or Latino

**Race**: ○ Caucasian ○ Native American ○ Black/AfricanAmerican ○ Asian ○ native Hawaiian or Pacific Islander

○ More than one race, not defined ○ Unknown or I prefer not to answer

**Jewish:** ○ Yes ○ No ○ Unknown **If yes:**  ○Ashkenazi ○Sephardic ○ Other Jewish Ancestry

*Maternal Grandfather:*  Where was he born (City/Country)?__________________________

**Ethnicity**: ○ Hispanic or Latino ○ Not Hispanic or Latino

**Race**: ○ Caucasian ○ Native American ○ Black/AfricanAmerican ○ Asian ○ native Hawaiian or Pacific Islander

○ More than one race, not defined ○ Unknown or I prefer not to answer

**Jewish:** ○ Yes ○ No ○ Unknown **If yes:**  ○Ashkenazi ○Sephardic ○ Other Jewish Ancestry

*Paternal Grandmother*: Where was she born (City/Country)?_________________________

**Ethnicity**: ○ Hispanic or Latino ○ Not Hispanic or Latino

**Race**: ○ Caucasian ○ Native American ○ Black/AfricanAmerican ○ Asian ○ native Hawaiian or Pacific Islander ○ More than one race, not defined ○ Unknown or I prefer not to answer

○ Asian ○ More than one race, not defined : _ Unknown or not reported

**Jewish:** ○ Yes ○ No ○ Unknown **If yes:**  ○Ashkenazi ○Sephardic ○ Other Jewish Ancestry

*Paternal Grandfather:*  Where was he born (City/Country)?___________________________

**Ethnicity**: ○ Hispanic or Latino ○ Not Hispanic or Latino

**Race**: ○ Caucasian ○ Native American ○ Black/AfricanAmerican ○ Asian ○ native Hawaiian or Pacific Island

○ More than one race, not defined : _ Unknown or not reported

**Jewish:** ○ Yes ○ No ○ Unknown **If yes:**  ○Ashkenazi ○Sephardic ○ Other Jewish Ancestry

**Birth Order**:  First  Second  Third  Other (specify: __________)

**Breast Fed:**  yes  no If yes, duration of exclusive breastfeeding ____Month ______Years____Unknown

**Delivery :**  Vaginal  C section  Unknown

**Date of Initial diagnosis: _____________ Age at diagnosis: ________ Date of Initial symptoms: ___________ mm/YYYY mm/YYYY**

**Current Diagnosis:  Ulcerative Colitis  Crohn’s Disease  Indeterminate**

**Initial Diagnosis:  Ulcerative Colitis  Crohn’s Disease  Indeterminate**

**If current diagnosis is different from initial diagnosis, date of change of diagnosis: ________________**

**Reasons for change of diagnosis (if applicable): __________________________ *mm/ yyyy***

How many No. Siblings__________No Children__________

**Family History:**

Do you have a family history of inflammatory bowel disease (including ulcerative colitis or Crohn’s disease)?

Yes ____ No______ I am not sure/I do not know_________

Please answer in the table below only if you have said “yes” to having a family history of inflammatory bowel **disease:**

|  | Number affected | Crohn’s disease | Ulcerative colitis | Unsure what type of inflammatory bowel disease | Age at time of diagnosis | Birth order  (i.e. 1^st^ born, 2^nd^ born, etc) |
| --- | --- | --- | --- | --- | --- | --- |
| Daughter |  |  |  |  |  |  |
| Son |  |  |  |  |  |  |
| Full Brother |  |  |  |  |  |  |
| Full Sister |  |  |  |  |  |  |
| Mother |  |  |  |  |  |  |
| Father |  |  |  |  |  |  |
| Maternal grandmother |  |  |  |  |  |  |
| Maternal grandfather |  |  |  |  |  |  |
| Paternal grandmother |  |  |  |  |  |  |
| Maternal grandfather |  |  |  |  |  |  |
| Paternal uncles |  |  |  |  |  |  |
| Maternal aunts |  |  |  |  |  |  |
| Maternal Cousin |  |  |  |  |  |  |
| Paternal Cousin |  |  |  |  |  |  |
| Other |  |  |  |  |  |  |
| Other |  |  |  |  |  |  |
| Other |  |  |  |  |  |  |

**PAST MEDICAL HISTORY**

Diabetes:  Yes  No  Unknown

Asthma:  Yes  No  Unknown

Multiple sclerosis:  Yes  No  Unknown

Systemic Lupus Erythematosus:  Yes  No  Unknown

History of delayed growth:  Yes  No  Unknown

Thyroid disease:  Yes  No  Unknown

Pulmonary Diagnosis  Yes  No  Unknown Must specify:________________

Neurological Diagnosis  Yes  No  Unknown Must specify::________________

Autoimmune Diagnosis  Yes  No  Unknown Must specify::________________

Other Medical conditions (specify): ___________________________________

**Cancer**

Diagnosis of colorectal dysplasia (precancer) /cancer:  Yes  No  Unknown

Surgery for colorectal dysplasia (precancer) /cancer:  Yes  No  Unknown

Diagnosis of other Cancer (specify)______________  Yes  No  Unknown

**Smoking History Prior to Diagnosis**   Cigarretes  Tobacco

Smoking at time of diagnosis:  Yes  Ex-smoker  No  Unknown

If Yes or Ex-smoker: Year started: ______ Year stopped: ______ No. of cigarettes per day: ____  Unknown

Do you Currently live with anyone who smokes inside your home ? (1 pack = 20 cigarettes)

 Yes  No  Unknown

**Alcohol History prior to Diagnosis**

Drinking at time of diagnosis:  Yes  Ex-drinker  No  Unknown

If Yes or Ex-drinker: Year started: _______Year stopped:_____ No. of drinks per day: ____  Unknown

**HAVE YOU USED CONTRACEPTIVES? (if applicable)**

 Yes  No

If yes, For how long?_____________ What type?_______________

**Menstrual History (if applicable)**

 Menstruating  Menopausal  Postmenopausal (over 1 year since last menstrual cycle)  Not Applicable or No Information

**Pregnancy History (if applicable)**

 None  Yes Number of births: ______ Date(s) of ALL delivery _______________________

**What insurance do you have:** No insurance Medicaid Medicare Group health plan Private insurance

**Level of education:**  Less than high school High school graduate Technical school College graduate

 Advanced degree (i.e. Master, Doctorate, PhD)  Other Refused  Unk / Not Provided

**Current marital status:**  Married  Divorced  Widowed  Separated  Never married

 A partner or unmarried couple  Refused

**SURGICAL HISTORY**

Have you had surgery for complications or treatment of IBD?  Yes  No  Unknown

If Yes, Which procedures have you had?

Common Crohn’s disease Surgeries: Date(s) Common Ulcerative Colitis surgeries: Date(s)

 Ileocolonic (small intestine and colon) Resection ______  Colon Resection (total) ”Pull-through” (J-Pouch) ____

 Small Intestine Resection ______  Total colon removal with ileostomy _____

 Colon Resection (partial) ______  Kock Pouch _____

 Diverting Ileostomy/Colostomy without total ______

colon removal (Circle which)

**Other Surgeries:** Date(s) Date(s)  Appendectomy ______  Fistulectomy/Fistulotomy ______

 Seton Placement ______  Mucosal advancement flap procedure ______

 Perirectal abscess draining ______  Perirectal abscess draining ______

 Strictureplasty

Fill in other surgeries not listed here: Date(s) Date(s)

 _______________________ ______  _______________________ ______

 _______________________ ______  _______________________ ______

**HOSPITALIZATION HISTORY (IBD-RELATED):  None Yes How many times_________**

**If** Yes, Reason:  Disease Flare (no surgery) Dates _____________

 Disease Flare (with surgery) Dates _____________

 Obstruction (Intestinal Blockage) Dates _____________

 Other Dates _____________

When was your last Flare (even if not required hospitalization? Date________ (m/d/y)

**Extra-Intestinal Manifestations**

**Eyes**:

Iritis *(difficulty seeing bright light):* ○ Yes ○ No ○ Unknown

Conjunctivitis: *(Inflammation of eyes with sticky deposit)* ○ Yes ○ No ○ Unknown

Uveitis *(sensitivity to light):* ○ Yes ○ No ○ Unknown

Episcleritis *(red eyes):* ○ Yes ○ No ○ Unknown

Undiagnosed eye inflammation: ○ Yes ○ No ○ Unknown

**Liver**:

Primary sclerosing cholangitis: ○ Yes ○ No ○ Unknown

*(Narrowing of bile ducts leading to cirrhosis)*

Hepatitis *(inflammation of liver):* ○ Yes ○ No ○ Unknown **If yes, which type of hepatitis?:** ○ A ○ B ○ C ○ Autoimmune

Other liver disease/abnormal liver function tests (**please specify)**: _____________________________________

**Skin**:

Erythema nodosum *(painful /red nodules):* ○ Yes ○ No ○ Unknown

Pyoderma gangrenosum *(ulcerated lesion):* ○ Yes ○ No ○ Unknown

Psoriasis *(Recurring reddish patches covered with silvery scales)*: ○ Yes ○ No ○ Unknown

**Joints**:

Arthritis associated with IBD ○ Yes ○ No ○ Unknown

*(Swelling, pain and reduction in flexibility of joints other than spine)*

Ankylosing spondylitis ○ Yes ○ No ○ Unknown

*(Pain* *and stiffness in joints of the spine and hip)*

Sacro-iliitis (*Inflammation of sacro-iliac lower spine joints)* ○ Yes ○ No ○ Unknown

Joint pain without swollen joints (*arthralgia)*: ○ Yes ○ No ○ Unknown

**Fistula**

Perianal *(opening around anus):* ○ Yes ○ No ○ Unknown

Rectovaginal *(tissue connection between the rectum and vagina)* ○ Yes ○ No ○ Unknown

Enteroenteric *(tissue connection between organs):* ○ Yes ○ No ○ Unknown

**If yes, please specify organs affected *(****i.e., loops of intestine; bladder)****:* ___________________**

**Fissure** *(crack in the skin around anus):* ○ Yes ○ No ○ Unknown

**Other**

Canker sores (*Aphthous Stomatitis)* ○ Yes ○ No ○ Unknown

Pouchitis ○ Yes ○ No ○ Unknown

Internal Abscess ○ Yes ○ No ○ Unknown

Perianal Abscess ○ Yes ○ No ○ Unknown

**Please complete the following questions regarding your medication history to the best of your ability:**

What medications for inflammatory bowel disease (Crohn’s or ulcerative colitis) are you taking at present:

Medication: _____________Dosage:___________ Frequency (how often?):_______ Last Doses_______

Start date: _____________ (if only remember year write 1/1/Year)

Medication: _____________Dosage:____________Frequency (how often?):_______Last Doses_________

Start date: _____________ (if only remember year write 1/1/Year)

Medication: _____________Dosage:____________Frequency (how often?):______ Last Doses_________

Start date: _____________ (if only remember year write 1/1/Year)

**Medication use History**

| Medication type and name | Prior or present use | Duration on medication (if taking at present please include the time you have been on medication until now)  **If only remember year please write 1/1/year** | Did you experience any severe side effects or allergic reactions? | Please specify side effect or reaction |
| --- | --- | --- | --- | --- |
| **Biologics** |  |  |  |  |
| Remicade (infliximab) | Yes____No______ | Start date________  End date ________ | Yes____No____ |  |
| Inflectra or Remsisa | Yes____No______ | Start date________  End date ________ | Yes____No___ |  |
| Humira (adalimumab) | Yes____No______ | Start date________  End date ________ | Yes____No____ |  |
| Cimzia (Certolizumab) | Yes____No______ | Start date________  End date ________ | Yes____No___ |  |
| Tysabry (natalizumab) | Yes____No______ | Start date________  End date ________ | Yes____No___ |  |
| Golimumab (Simponi) | Yes____No______ | Start date________  End date ________ | Yes____No___ |  |
| Ustekinumab (Stelara) | Yes____No______ | Start date________  End date ________ | Yes____No___ |  |
| Vedolizumab (Entyvio) | Yes____No______ | Start date________  End date ________ | Yes____No___ |  |
| Other: | Yes____No______ | Start date________  End date ________ | Yes____No___ |  |
| **Small molecules** |  |  |  |  |
| Tofactinib (Xeljanz) | Yes____No______ | Start date________  End date ________ | Yes____No___ |  |
| Other: | Yes____No______ | Start date________  End date ________ | Yes____No___ |  |
| **Immunomodulator** |  |  |  |  |
| 6-mp (mercaptopurine) | Yes____No______ | Start date________  End date ________ | Yes____No___ |  |
| azathioprine (Imuran) | Yes____No______ | Start date________  End date ________ | Yes____No___ |  |
| Cyclosoporine | Yes____No______ | Start date________  End date ________ | Yes____No___ |  |
| Methotrexate | Yes____No______ | Start date________  End date ________ | Yes____No___ |  |
| Tacrolimus | Yes____No______ | Start date________  End date ________ | Yes____No___ |  |
| Other: | Yes____No______ | Start date________  End date ________ | Yes____No___ |  |
| **Medication type and name** | **Prior or present use** | **Duration on medication (if taking at present please include the time you have been on medication until now)** | **Did you experience any severe side effects or allergic reactions?** | **Please specify side effect or reaction** |
| **Mesalamines** |  |  |  |  |
| Asacol | Yes____No______ | Start date________  End date ________ | Yes____No___ |  |
| Apriso | Yes____No______ | Start date________  End date ________ | Yes____No___ |  |
| Pentasa | Yes____No_____ | Start date________  End date ________ | Yes____No__ |  |
|  |  |  |  |  |
| Sulfasalazine | Yes____No_____ | Start date________  End date ________ | Yes____No______ |  |
| Colazal | Yes____No_____ | Start date________  End date ________ | Yes____No______ |  |
| Lialda | Yes____No_____ | Start date________  End date ________ | Yes____No______ |  |
| **Mesalamine via rectum (suppositories or enemas)Rectal Enemas/suppositories** |  |  |  |  |
| Rowasa Enema | Yes____No_____ | Start date________  End date ________ | Yes____No______ |  |
| Canasa suppository | Yes____No_____ | Start date________  End date ________ | Yes____No______ |  |
| Cortisone Enema | Yes____No_____ | Start date________  End date ________ | Yes____No______ |  |
| Other___________ | Yes____No_____ | Start date________  End date ________ | Yes____No______ |  |
| **Corticosteroids** |  |  |  |  |
| Prednisone | Yes____No_____ | Start date________  End date ________ | Yes____No______ |  |
| Entocort | Yes____No_____ | Start date________  End date ________ | Yes____No______ |  |
|  | Yes____No_____ |  |  |  |
| Other___________ | Yes____No_____ | Start date________  End date ________ | Yes____No______ |  |

**Steroid use (includes prednisone, uceris, budesonide (entocort):**

How many times in the past year have you been on a steroid?______________

how many times since diagnosis have you been on any steroid? ____________

what is the longest you have ever been on a steroid?___________

**Antibiotics History:**

Have you ever been placed on antibiotics for treatment of crohn’s disease o ulcerative colitis? Yes_____No_____

how many times? ____

**Oral pain medication (i.e percocet, oxycodone, morphine, dilaudid)** to help with pain from crohn’s disease or Colitis Ulcerative ? yes____No_____If yes, please specificy type: ________________

Are you currently taking oral pain medications? Yes ____ No_____ How long have you been on them? ___________

**Do you take any herbal products or supplements for your inflammatory bowel disease**? Yes_________ No___________

Please specify what type of product you take (for example, curcumin/turmeric)?_____________________

For how long? ______________________

**Have you used marijuana or cannabinoids or CBD oil for your inflammatory bowel disease**? Yes________No__________

Please specify what type of product you take ________________________For how long? _________

Are you currently taking any ? yes____ No__Have you felt improvement of your symptoms with cannabinoids? Yes_______ No_________ Not applicable____

**Environmental History**

|  | 1 yr old | 2-4 yrs | 5-15 yrs | Age Range  16-24 yrs | | 25-30 yrs | Currently |
| --- | --- | --- | --- | --- | --- | --- | --- |
| Not Applicable |  |  |  | |  |  |  |
| No Pets |  |  |  | |  |  |  |
| Cat |  |  |  | |  |  |  |
| Dog |  |  |  | |  |  |  |
| Bird |  |  |  | |  |  |  |
| Turtle |  |  |  | |  |  |  |
| Rabbit |  |  |  | |  |  |  |
| Hamster |  |  |  | |  |  |  |
| Guinea Pig |  |  |  | |  |  |  |
| Fish |  |  |  | |  |  |  |
| Rat |  |  |  | |  |  |  |
| Mice |  |  |  | |  |  |  |
| Other (please specify) |  |  |  | |  |  |  |
| Other (please specify) |  |  |  | |  |  |  |

**Pet History**

**Physical Activities**

Are activities where you move and increase your heart rate above its resting rate, whether you do them for pleasure, work, or transportation? The following questions ask about the amount and intensity of physical activity you usually do. Examples of physical activity intensity levels:

**Light Activities** Your heart beats slightly faster than normal**;** you can talk and sing

(Walking Leisurely, Stretching, Light Yard Work)

**Moderate Activities** Your heart beats faster than normal You can talk but not sing (Fast Walking, Aerobics Class, Strength, swimming

**Vigorous Activities** Your heart rate increases a lot You can't talk or your talking is broken up by large breath

(Stair Machine, Jogging or Running, Tennis, Racquetball, Pickle ball or Badminton)
**1. What best describes your activity level?**

 vigorously active for at least 30 min, 3 times per week

 moderately active at least 3 times per week

 seldom active, preferring sedentary activities

**2. Compared to other people your own age, do you think you are**

 much more active,

 more active

 about as active

 less active

 much less active

Please answer questions below **only if you are Hispanic or Latino** and **were born outside of the US.**

**Language:**

In general, what language (s) do you speak and read?

Only English _____English better than Spanish_____ Both equally____Spanish better than English_____Only Spanish_____

What language do you speak usually at home?

Only English _____English better than Spanish_____ Both equally____Spanish better than English_____Only Spanish_____

In what language do you usually think?

Only English _____English better than Spanish_____ Both equally____Spanish better than English_____Only Spanish_____

What language do you usually speak with your friends?

Only English _____English better than Spanish_____ Both equally____Spanish better than English_____Only Spanish_____

**Environmental History**

**This section will ask about your environment and what you were exposed to as a child, teenager and as an adult. Please answer to the best of your ability**

|  | **During your first year of life and up to 5 years** | **From 6 to 18 years old** | **Greater than 18 years old** | **Before your diagnosis** | **After your diagnosis** |
| --- | --- | --- | --- | --- | --- |
| Did you live on a farm or visit a farm on a daily basis? | Yes____  No_____  Unsure_____ | Yes____  No_____  Unsure_____ | Yes____  No_____  Unsure____  I am not an greater than 18________ | Yes____  No_____  Unsure_____ | Yes____  No_____  Unsure_____ |
| **If yes, what type of farm**? |  |  |  |  |  |
| Dairy cattle | Yes___No___ | Yes___No____ | Yes___No____ | Yes___No____ | Yes___No____ |
| Beef cattle | Yes___No____ | Yes___No____ | Yes___No____ | Yes___No____ | Yes___No____ |
| Chicken | Yes___No____ | Yes___No____ | Yes___No____ | Yes___No____ | Yes___No____ |
| Grain/wheat | Yes___No____ | Yes___No____ | Yes___No____ | Yes___No____ | Yes___No____ |
| Other | Yes___No____ | Yes___No____ | Yes___No____ | Yes___No____ | Yes___No____ |
| **What was your main source of water?** | Private well____  Community supply_______  Bottle water_______  Other_______  Unsure______ | Private well____  Community supply_______  Bottle water_______  Other_______  Unsure______ | Private well____  Community supply_______  Bottle water_______  Other_______  Unsure______ | Private well____  Community supply_______  Bottle water_______  Other_______  Unsure______ | Private well____  Community supply_______  Bottle water_______  Other_______  Unsure______ |
| **Did you routinely drink unpasteurized milk?** | Yes ____  No _____  Unsure_____ | Yes____  No_____  Unsure_____ | Yes____  No_____  Unsure____  I am not an greater than 18______ | Yes____  No_____  Unsure_____ | Yes____  No_____  Unsure_____ |
| **How many people lived in your home including you?** | Less than 3___  Less than 5___  Less than 8___  8 or more ___  Don’t know | Less than 3___  Less than 5___  Less than 8___  8 or more ___  Don’t know | Less than 3___  Less than 5___  Less than 8___  8 or more ___  Don’t know | Less than 3___  Less than 5___  Less than 8___  8 or more ___  Don’t know | Less than 3___  Less than 5___  Less than 8___  8 or more ___  Don’t know |
| **How many bathrooms were there in your home?** | One____  Two____  Three___  More___  Don’t know____ | One____  Two____  Three___  More___  Don’t know____ | One____  Two____  Three___  More___  Don’t know____ | One____  Two____  Three___  More___  Don’t know____ | One____  Two____  Three___  More___  Don’t know____ |
| **Did you receive antibiotics? More than 3 times in a year** | Yes____  No_____  Unsure_____ | Yes____  No_____  Unsure_____ | Yes____  No_____  Unsure_____ | Yes____  No_____  Unsure_____ | Yes____  No_____  Unsure_____ |
| **Did you get diagnosed or treated for parasitic infections?** | Yes____  No_____  Unsure_____ | Yes____  No_____  Unsure_____ | Yes____  No_____  Unsure____  I am not an greater than 18______ | Yes____  No_____  Unsure_____ | Yes____  No_____  Unsure_____ |
| **Were you exposed to non-steroidal anti-inflammatories (ibuprofen, aleve, naproxen)** | Yes____  No_____  Unsure_____ | Yes____  No_____  Unsure_____ | Yes____  No_____  Unsure____  I am not an greater than 18______ | Yes____  No_____  Unsure_____ | Yes____  No_____  Unsure_____ |

**The Following Section To Be Completed By Physician**

****note: please base all assessments on *pre-surgical* conditions, when applicable****

*Phenotype assessments should reflect the most severe episode a patient has ever experienced.*

**Disease Location (Microscopic extent supersedes Macroscopic extent)**

○ Oropharyngeal ○ Esophagus ○ Stomach ○ Duodenum ○ Jejunum ○ Ileum

○ Cecum ○ Asc. Colon ○ Transv. Colon ○ Desc. Colon ○ Sigmoid ○ Rectum

**Diagnosed with**

○ Crohns ○ Ulcerative Colitis ○ Indetermine

**Crohn’s disease behavior**

Fibrostenotic ○ Yes ○ No ○ Unknown

Inflammatory ○ Yes ○ No ○ Unknown

Internal penetrating/perforating ○ Yes ○ No ○ Unknown

Perianal perforating ○ Yes ○ No ○ Unknown

UC-like ○ Yes ○ No ○ Unknown

**Ulcerative Colitis severity** *(classify by most severe past disease episode)*

○ S0 (Clinical Remission, Asymptomatic)

○ S1 (Mild UC, ≤4 stools/day (± blood), no systemic illness, normal inflammatory markers (ESR))

○ S2 (Moderate UC, ≥4 stools/day, minimal systemic toxicity)

○ S3 (severe UC, ≥6 bloody stools/day, pulse rate ≥90 bpm, temp. ≥37.5^o^C, hemoglobin <10.5g/100 ml, ESR ≥30mm/h)

**Cancer History**

History of dysplasia : Yes___ No ____ IBD-associated ? Yes____No____ Unclear_______

Surgery for dysplasia or colorectal cancer?  Yes  No  Unclear

Other history of cancers?  Yes  No  Unknown. Type: ___________________
